# Development and Pilot Evaluation of a Wearable 12-Lead ECG System for Multilead Feature Analysis in Individuals with Different Glycemic Status

**DOI:** 10.3390/s26051598

**Published:** 2026-03-04

**Authors:** Chingiz Alimbayev, Zhadyra Alimbayeva, Kassymbek Ozhikenov, Kairat Karibayev, Zhansila Orynbay, Yerbolat Igembay, Madiyar Daniyalov, Akzhol Nurdanali

**Affiliations:** 1 Joldasbekov Institute of Mechanics and Engineering, Almaty 050010, Kazakhstan; c.alimbayev@satbayev.university (C.A.); karibayev@snh.kz (K.K.); z.orynbay@satbayev.university (Z.O.); y.igembay@satbayev.university (Y.I.); a.nurdanali@satbayev.university (A.N.); 2 Department of Robotics and Technical Means of Automation, Satbayev University, Almaty 050013, Kazakhstan; 3 Department of Information Technology and Library Science, Kazakh National Women’s Teacher Training University, Almaty 050000, Kazakhstan; 4 Department of Disaster Medicine and Emergency Care Organization, Kazakh-Russian Medical University, Almaty 050013, Kazakhstan

**Keywords:** wearable ECG device, multilead electrocardiography, ventricular repolarization, glycemic status, prediabetes, type 2 diabetes mellitus, ECG signal, digital health monitoring, non-invasive metabolic screening, electrophysiological biomarkers

## Abstract

Type 2 diabetes mellitus and prediabetes often develop silently and may remain undiagnosed for years. This is particularly relevant in regions where laboratory-based screening is not always readily accessible. Against this background, the present work explores whether multilead electrocardiography can provide physiologically meaningful markers potentially associated with disturbances in glucose metabolism. We developed and tested an upgraded wearable 12-lead ECG system capable of synchronized multichannel recording under controlled conditions. ECG signals were acquired in sitting and standing positions, with a sampling frequency of 500 Hz and a recording duration of one minute per posture. The hardware architecture included a high resolution analog front-end and wireless data transmission; the accompanying software provided acquisition control, preprocessing, visualization, and data storage within a unified framework. Signal processing focused on the extraction of rhythm-related and morphological parameters, with particular attention to ventricular repolarization indices. QT interval, heart rate–corrected QT (QTc), and QT dispersion (QTd) were calculated across leads, as these parameters are known to reflect heterogeneity of repolarization and autonomic influences on myocardial electrophysiology. The analysis was structured to ensure reproducible boundary detection and systematic feature formation rather than isolated parameter measurement. The study had a pilot character and included a limited and unbalanced sample (healthy n = 10; prediabetes n = 1; T2DM n = 1). For this reason, the results are presented descriptively and should be regarded as preliminary observations. In representative cases, differences in QT-related indices were noted between categories of glycemic status; however, the potential influence of age, sex, and other confounders cannot be excluded. A pilot expert comparison of T-wave end detection demonstrated close agreement between the automated algorithm and cardiologist assessment (mean Δ$T_{end}$ approximately −1 to −2 ms; MAE 10–24 ms). Diagnostic performance metrics such as ROC/AUC, sensitivity, and specificity were not calculated at this stage, as validation in a larger cohort with biochemical confirmation (HbA1c, OGTT) is required. The study demonstrates the technical feasibility of combining synchronized 12-lead wearable acquisition with structured multilead repolarization analysis. The proposed system should therefore be considered a research platform intended to support further clinical validation and methodological development rather than a finished screening solution.

## 1. Introduction

Type 2 diabetes mellitus (T2DM) is one of the most prevalent chronic diseases worldwide and represents a major challenge for healthcare systems. By 2045, the global number of individuals with diabetes is expected to reach approximately 700 million, while nearly half of affected adults remain unaware of their condition [1]. The majority of patients live in low- and middle-income countries, where access to early diagnosis and preventive care is often limited. Undiagnosed T2DM frequently leads to severe complications, particularly cardiovascular disorders. In Kazakhstan, the reported prevalence of T2DM ranges from 8.2% to 12.5% [2]. Despite the implementation of national screening programs, a substantial proportion of the population continues to exhibit major risk factors, including overweight, which affects approximately 54% of citizens, and elevated cholesterol levels, observed in about 45%.

Diabetes mellitus is generally classified into two main types, with more than 90% of all cases being T2DM [3]. T2DM develops gradually over a period of 10–20 years and is primarily associated with impaired glucose utilization due to reduced tissue sensitivity to insulin, known as insulin resistance [4]. Even at the prediabetes stage, insulin sensitivity is already diminished, resulting in altered glucose metabolism at the tissue level. Studies indicate that the earliest pathological changes may occur decades before the clinical manifestation of the disease, during which long-term metabolic disturbances are established. It is estimated that up to 60% of individuals with prediabetes eventually progress to T2DM [5].

Early identification of prediabetes is therefore considered a key preventive strategy, as it enables timely lifestyle interventions, regular medical monitoring, and a reduction in the risk of progression to overt diabetes. The American Diabetes Association recommends population screening for the detection of prediabetes [6]. In clinical practice, fasting plasma glucose testing [7] and the oral glucose tolerance test [8] are commonly used. Additional approaches include measurements of fasting insulin levels, the hyperinsulinemic euglycemic clamp, and derived indices [9]. However, these methods are characterized by high interlaboratory variability and the absence of a single universally accepted “gold standard”, which limits their reliability and feasibility for large-scale screening.

Alternative strategies based on biochemical biomarkers have also been proposed. For example, the assessment of 2-hydroxybutyrate levels [10], along with other complementary markers [11,12], has been used to estimate T2DM risk by comparison with reference values. Such approaches allow for the classification of individuals as having normal glucose tolerance, impaired glucose tolerance, or diabetes. In addition, biochemical parameters such as lipid profiles, glycated hemoglobin (HbA1c), C-peptide, and adiponectin [13], as well as anthropometric data, may be incorporated into predictive mathematical models [14]. Although these methods can achieve high accuracy, they remain complex, require laboratory infrastructure, and are therefore poorly suited for rapid and widespread screening.

Another actively developing direction involves big data analysis, which integrates medical imaging, genetic information, biochemical markers, and lifestyle data [15,16]. Advanced computational techniques, including deep learning [17], genetic algorithms [18], and recurrent neural networks [19], are employed to extract informative features, construct predictive models based on random and causal forests, and generate personalized prevention strategies. Nevertheless, these approaches require substantial computational resources and expert interpretation, limiting their applicability outside specialized centers.

Diabetes mellitus exerts a pronounced impact on the cardiovascular system. Metabolic disturbances contribute to the development of diabetic cardiomyopathy [20], which is characterized by structural and functional myocardial alterations independent of arterial hypertension or ischemic heart disease. Diabetic neuropathy plays a critical role by affecting autonomic cardiac regulation and causing an imbalance between sympathetic and parasympathetic activity [21]. Chronic hyperglycemia and increased blood viscosity impair microcirculation, leading to persistent myocardial ischemia and fibrosis [22,23]. These processes increase the risk of arrhythmias, sudden cardiac death, and heart failure in patients with T2DM.

A range of diabetes-related alterations can be detected using electrocardiography. Electrocardiographic abnormalities are observed in approximately 25% of patients with T2DM who do not present overt clinical symptoms. Common findings include nonspecific ST–T segment changes, signs of left ventricular hypertrophy, and impaired contractile function of the anterior left ventricular wall [24]. In patients with T2DM, prolongation of the Tp–e interval as well as increased Tp–e/QT and Tp–e/QTc ratios has been reported [25], indicating enhanced transmural dispersion of repolarization and an elevated risk of ventricular arrhythmogenesis. According to a previous study [26], even at early stages of diabetes, ECG alterations such as sinus tachycardia, QTc prolongation, increased QT dispersion, reduced heart rate variability, ST–T changes, and left ventricular hypertrophy may be observed.

Therefore, electrocardiography can be regarded as a low-cost, non-invasive, and widely accessible tool for the early detection of diabetes-related complications. In recent years, increasing attention has been directed toward the use of ECG not only for diagnosing established cardiac pathology but also for identifying early metabolic alterations associated with prediabetes [27]. The emergence of advanced signal processing and machine learning techniques enables the extraction of latent features from high-frequency and morphological ECG characteristics that were previously not utilized in routine clinical practice [28].

In this context, investigating the relationship between ECG features and metabolic disturbances in T2DM is of particular interest, as it opens new possibilities for screening and monitoring applications. The development of specialized portable ECG devices equipped with intelligent analysis algorithms may support future large-scale validation and screening-oriented applications, especially in countries with limited healthcare resources, such as Kazakhstan.

The aim of this study is to explore the feasibility of identifying diabetes and prediabetes based on electrocardiographic characteristics potentially associated with metabolic disturbances. To achieve this objective, we upgraded a previously proposed wearable ECG device [29,30], originally designed for cardiac monitoring and diagnostics. In earlier studies, the system operated in a single-lead configuration (four channels), whereas the present work introduces an upgraded prototype with extended acquisition capabilities, supporting 12 leads in a 10-electrode configuration.

Although ECG-based detection of dysglycemia has been explored in recent years, most prior studies have relied on retrospective databases, single-lead recordings, or pre-extracted ECG features, without addressing the design of the acquisition system itself [31,32]. Consequently, limited attention has been paid to the development of a dedicated wearable multilead platform specifically optimized for electrophysiological markers potentially associated with metabolic disturbances.

In this study, we present an integrated approach that combines synchronized 12-lead wearable ECG acquisition, real-time signal quality control, and a structured algorithm for extracting multilead repolarization indices, including QT dispersion. Rather than applying purely data-driven models to existing datasets, our work emphasizes physiologically interpretable ECG markers and controlled signal acquisition conditions, forming a coherent acquisition-to-analysis pipeline for feasibility research in non-invasive metabolic screening.

The novelty of the present work lies not in the hardware configuration itself, but in the structured extraction of multilead repolarization heterogeneity indices using a wearable acquisition platform.

## 2. Materials and Methods

### 2.1. Hardware Development of Device

**Figure 1:**
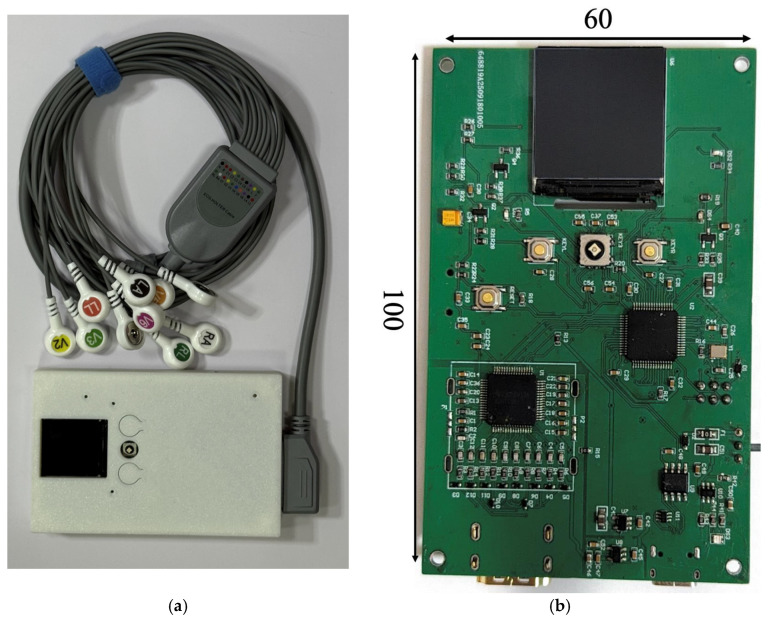
Photographs of the upgraded wearable ECG device (**a**) and its printed circuit board (**b**).

Photographs of the wearable ECG device developed by the authors and its printed circuit board (PCB) are shown in Figure 1.

The developed electrocardiograph is a multichannel wearable device with compact dimensions of 100 × 60 mm, enabling the acquisition of 12 standard ECG leads using ten electrodes arranged according to the conventional clinical placement scheme.

**Figure 2:**
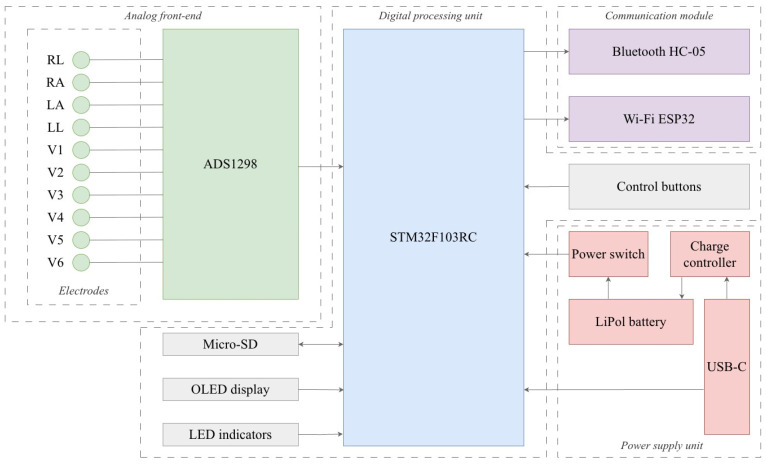
Functional block diagram of the ECG device.

Figure 2 illustrates a functional block diagram of the ECG system.

The signal acquisition core of the system is based on the ADS1298 analog-to-digital converter (Texas Instruments, Dallas, TX, USA), featuring a 24-bit resolution and a sampling rate of at least 1000 Hz. In this study, ECG signals were recorded and analyzed at Fs = 500 Hz. The architecture supports the synchronous operation of multiple ADC chips to enable the simultaneous recording of all ten channels. Data transmission during the study was performed via the integrated Wi-Fi module (ESP32, Shanghai, China) using a local TCP/IP connection within a controlled laboratory network. The Bluetooth HC-05 module (Guangzhou HC Information Technology Co., Ltd., Guangzhou, China) was included for auxiliary debugging and serial communication during development but was not used for ECG streaming in the reported experiments. In addition, a USB-C port is implemented for device programming, device firmware update (DFU) mode, and battery charging. The device is equipped with a microSD card slot (up to 32 GB, FAT32) for the local storage of raw ECG data and a compact OLED display (23 × 23 mm, resolution ≥ 128 × 64 pixels) for the real-time visualization of ECG waveforms, heart rate, connection status, and battery level. Power is supplied by a 2000 mAh (3.7 V) lithium-ion battery (Shenzhen EEMB Battery Co., Ltd., Shenzhen, China) with a TP4056 charging module, providing a minimum of eight hours of autonomous operation.

The presented electrical schematic incorporates an STM32F103RC microcontroller (STMicroelectronics, Geneva, Switzerland), to which control, clock, and digital interface lines are connected to ensure communication with peripheral modules. The analog front-end is implemented using the ADS1298 integrated circuit, which is connected to the electrode inputs through matching and filtering networks. Power supply and reference voltages are provided via dedicated stabilized lines, while communication with the microcontroller is performed over the SPI interface using dedicated DIN, DOUT, SCLK, and CS signal lines.

The data storage subsystem is implemented via a microSD card slot connected to the microcontroller through the SPI bus, with appropriate power conditioning and noise decoupling. Wireless communication modules, including Bluetooth HC-05 and Wi-Fi ESP32, feature independent power supply circuits with RC input filtering and are interfaced with the main controller via UART and auxiliary GPIO lines. The OLED display (based on SSD1306 controller, Solomon Systech Ltd., Hong Kong, China) is connected via the I^2^C bus together with light-emitting indicators, each equipped with current-limiting resistors in its anode circuits.

The power management system comprises a lithium-polymer battery protected by charge and discharge control circuits, a TP4056-based charging module (Nanjing Top Power ASIC Corp., Nanjing, China), and a USB-C input equipped with filtering components, electrostatic discharge (ESD) protection, and 5 V power lines. Voltage regulators and decoupling capacitors are included to generate stable 3.3 V and 5 V supply rails and to suppress high-frequency noise across all digital and analog blocks. The overall circuit layout is complemented by grounding, filtering, and impedance-matching measures to ensure reliable operation of the high-resolution analog-to-digital conversion chain and peripheral modules. The complete component-level electrical schematic is provided in the Supplementary Materials (Figure S1). The main text focuses on the system architecture and functional structure, while the full detailed circuit diagram is available for technical reference.

**Figure 3:**
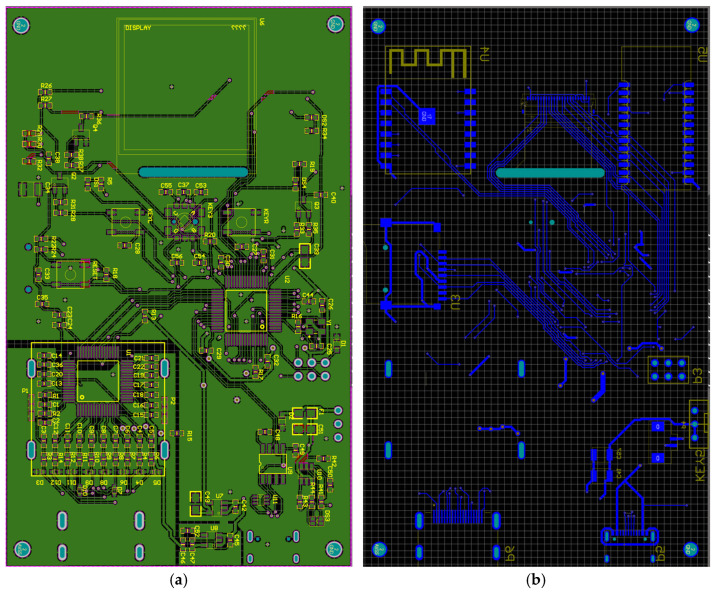
Printed circuit board layout of the device: (**a**) top layer; (**b**) bottom layer. Green—PCB substrate; red—top copper traces; blue—bottom copper traces; yellow—component silkscreen and outlines; cyan—mechanical holes and vias; purple—board outline; grey grid—design workspace reference.

Based on the developed electrical schematic, a two-layer printed circuit board was designed to accommodate all functional units of the biosignal acquisition device (Figure 3).

The top layer of the printed circuit board (Figure 3a) contains the main integrated circuits and high-frequency digital interfaces, including the STM32F103RC microcontroller, the ADS1298 analog front-end, and the SPI, UART, and I^2^C signal lines. These components are arranged to minimize trace lengths and reduce crosstalk. The bottom layer (Figure 3b) is primarily used for power routing and the formation of continuous ground planes, as well as for placing less noise-sensitive components such as the Bluetooth and Wi-Fi wireless modules, the microSD card slot, selected filtering capacitors, and power stabilization elements. Power traces are implemented with increased width to reduce electrical resistance and thermal losses. The analog section of the PCB includes the electrode input circuits and the supporting components of the ADS1298 and is physically isolated from the digital blocks. In this region, signal traces are kept as short as possible, while the grounding structure and applied shielding techniques contribute to effective noise reduction.

### 2.2. Electrode Placement and Lead Acquisition Principles

**Figure 4:**
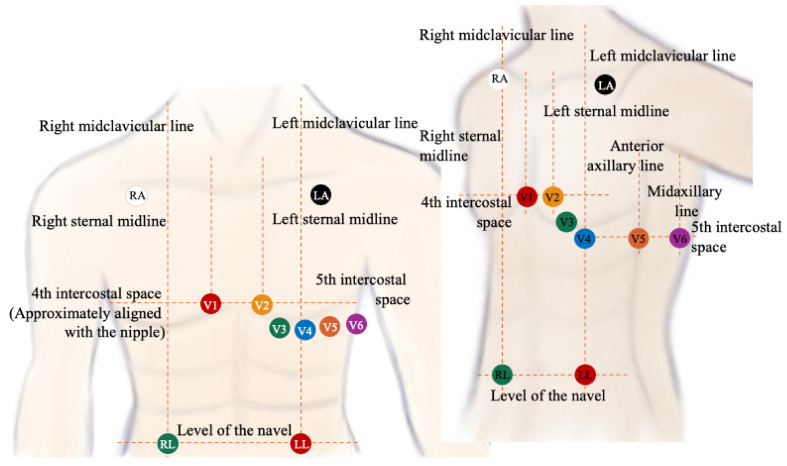
Standard anatomical placement of the ten electrodes required to obtain twelve conventional electrocardiographic leads.

Figure 4 illustrates the standard anatomical placement of ten electrodes required to obtain twelve conventional electrocardiographic leads.

The schematic depicts the placement of the limb electrodes (RA, LA, RL, and LL) and the six precordial electrodes (V1–V6) with respect to key anatomical landmarks of the thorax, including the right and left sternal borders, the midclavicular lines, the anterior axillary line, and the intercostal spaces. The precordial electrodes V1 and V2 are positioned in the fourth intercostal space along the right and left sternal borders, respectively, whereas electrodes V3, V4, V5, and V6 are arranged along the anterior chest wall, following the anatomical curvature of the ribs from the midclavicular to the midaxillary line. The limb electrodes are shown at the distal regions of the body, with RA and LA placed on the upper torso below the clavicles, and RL and LL positioned on the lower abdomen near the umbilical level.

Such graphical representation ensures unambiguous visualization of electrode placement, which is critical for achieving accurate and reproducible ECG acquisition, minimizing signal artifacts, and maintaining compliance with international clinical standards, such as the recommendations of the American Heart Association (AHA) and the American College of Cardiology Foundation (ACCF).

Table 1 presents the mathematical formulation of the twelve standard electrocardiographic leads derived from ten physical electrodes.

The table lists the equations used to calculate the Wilson central terminal (WCT), the standard bipolar limb leads (I, II, and III), the augmented unipolar limb leads (aVR, aVL, and aVF), and the six precordial (chest) leads referenced to the WCT. Each lead is defined as a specific linear combination of the measured electrode potentials, in accordance with the classical principles established by Einthoven and Goldberger. The table illustrates how the three limb electrodes (RA, LA, and LL) are used to construct both bipolar and augmented unipolar limb leads, whereas the precordial leads (V1–V6) are obtained by subtracting the WCT from the corresponding chest electrode potentials.

### 2.3. Software Development of Device

The software component of the device is implemented according to a modular architecture, providing sequential acquisition of data from the analog-to-digital converter, signal preprocessing, visualization, and storage. The software is developed using the Python (version 3.10) programming language, with the graphical user interface implemented in PyQt5 (version 5.15.10). Real-time signal visualization is performed using the pyqtgraph library, which enables efficient rendering of ECG waveforms. Mathematical signal processing is carried out using the NumPy and SciPy libraries, including vectorized computations, digital filtering, and statistical analysis. Communication with the hardware subsystem is organized via a UART interface using the pySerial module.

ECG signals are stored in a local SQLite relational database, where all data are maintained within a single portable file. For remote data exchange and result sharing, the gspread and oauth2 libraries are employed, enabling direct data export to Google Sheets.

**Figure 5:**
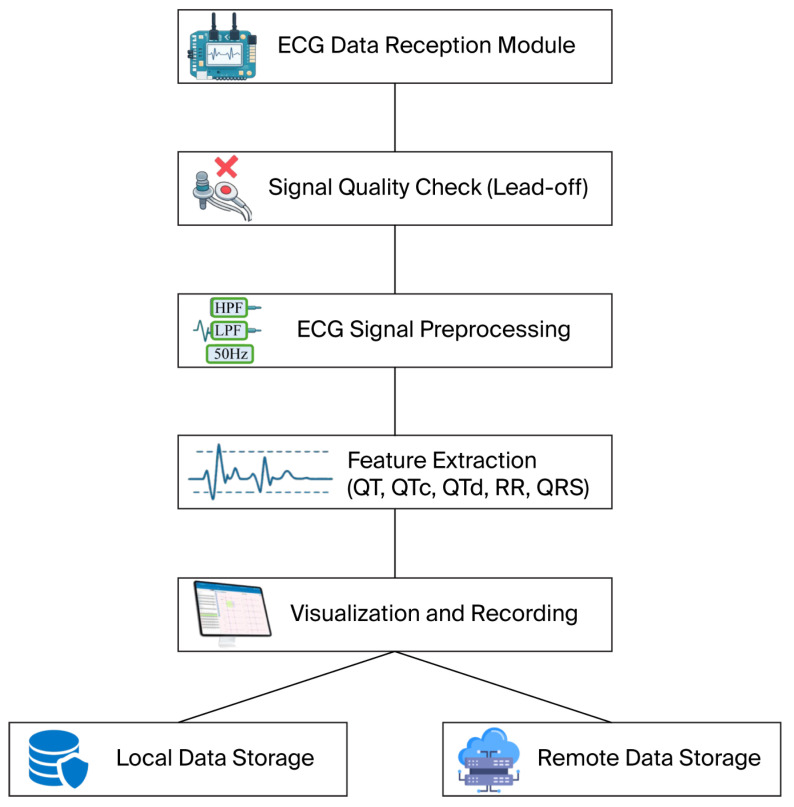
Modular software architecture and an ECG data-processing pipeline implemented in the proposed device.

The modular software block diagram (see Figure 5) illustrates a sequential vertical structure of ECG signal processing stages, ranging from data acquisition to visualization and long-term storage. This stepwise processing pipeline ensures clear functional separation, facilitates debugging, and enables straightforward system scalability.

Importantly, the software continuously monitors electrode status. Hardware lead-off bytes indicate loss of contact for individual leads, and when a disconnection is detected, data recording is automatically paused. These measures ensure that only reliable signals are included in the analysis, while disconnected channels are excluded from further processing.

ECG data are transmitted from the device in fixed-length packets of 40 bytes. The overall packet structure is summarized in Table 2. Each packet begins with a 3-byte synchronization header, followed by a payload length byte (34 bytes), 32 bytes containing raw data from eight ECG channels (I, II, and V1–V6) encoded as 32-bit integers (little-endian), 2 bytes of lead-off status flags, and 2 bytes of checksum values. This data organization enables reliable synchronization between the transmitter and receiver, separation of payload and control information, and verification of channel integrity.

### 2.4. Development of an ECG-Based Algorithm for the Assessment of Glycemic Status

The algorithm for glycemic status assessment is based on a stepwise processing of multichannel ECG signals, followed by the extraction of rhythm-related, morphological, and repolarization features that are potentially associated with metabolic alterations. The algorithm is implemented as a modular data-processing pipeline, in which each stage performs a well-defined function, ranging from ECG acquisition to the formation of a set of analysis parameters. This approach ensures processing reproducibility, computational transparency, and the possibility of further algorithm extension.

**Figure 6:**
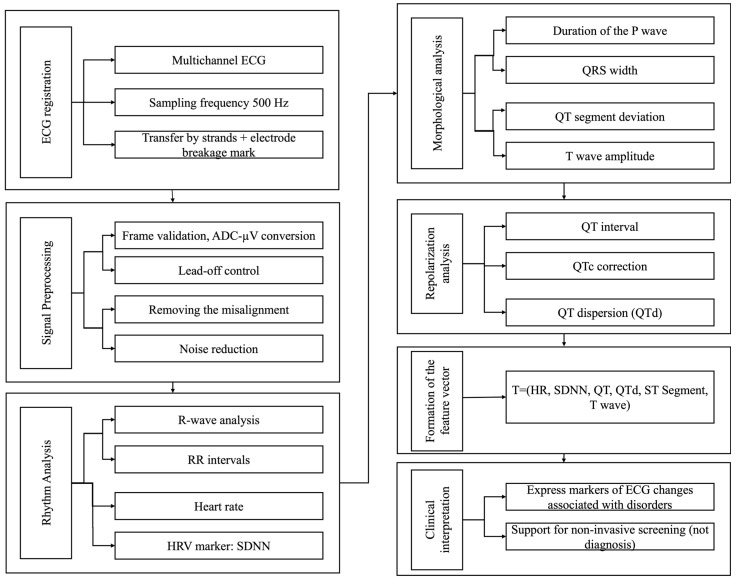
Stepwise modular scheme of the ECG signal processing algorithm for extracting features potentially associated with glycemic disturbances.

The overall algorithm structure and the sequence of the ECG signal processing stages are presented in Figure 6.

As shown in Figure 6, the algorithm begins with the acquisition of multichannel ECG signals at a sampling rate of 500 Hz. Data transmission is performed in a packet-based format with simultaneous registration of electrode lead-off flags, enabling real-time signal quality control during data collection.

At the next stage, ECG signal preprocessing is carried out, including frame validation, conversion of analog-to-digital converter values into physical units (microvolts), lead-off detection, baseline wander removal, and noise suppression. This stage aims to improve the signal-to-noise ratio and to exclude distorted signal segments from subsequent analysis.

Following preprocessing, heart rhythm analysis is performed, comprising R-peak detection, RR interval computation and heart rate estimation. These parameters reflect the state of autonomic cardiac regulation and are used as part of the feature vector. Given the 1 min recordings, reliable HRV assessment was not the primary endpoint and will be considered using longer recordings in future studies.

Subsequently, morphological ECG analysis is conducted, including measurements of P-wave duration, QRS complex width, ST-segment deviation, and T-wave amplitude. These parameters characterize myocardial depolarization and repolarization processes and may be altered in metabolic and ischemic conditions.

A dedicated stage is devoted to ventricular repolarization analysis, which includes calculation of the QT interval, heart rate-corrected QT (QTc), and QT dispersion (QTd), reflecting the heterogeneity of myocardial repolarization. The QT interval was defined as the time from the onset of the Q wave to the end of the T wave. QTc was calculated using the Bazett correction formula, while QT dispersion (QTd) was determined as the difference between the maximum and minimum QT values measured across standard ECG leads.

In the present implementation, QTd is calculated from leads I, II, and V1–V6. The lead is included in the calculation if there is a sufficient number of valid cycles (N ≥ 30 or ≥50% of all cycles in the analyzed 1 min fragment). Individual cycles are excluded if the amplitude of the T-wave is insufficient relative to the noise level, the morphology is ambiguous (suspicion of a U-wave or a two-phase T-wave), or the end of the T-wave ($T_{end}$) cannot be reliably determined. QT is measured for each cycle. Before calculating the final value for each lead, robust outlier filtering (MAD/IQR) is applied, after which the median of valid QT values is used as a stable estimate of QT_lead_. Next, QTd is calculated as the difference between max(QT_lead_) and min(QT_lead_) among the leads that meet the quality criteria (eligible leads). Additionally, the minimum threshold for the number of eligible leads (K ≥ 6) is set. If it is not met, QTd is marked as unavailable (NA) to exclude calculations based on insufficiently reliable data.

The QTc and QTd indices are considered key features potentially associated with alterations in glycemic status and an increased risk of arrhythmias. We used Bazett’s formula so that the results could be compared with previous work. It should be borne in mind that this method tends to overestimate the QTc with a frequent pulse and underestimate it with a rare one. Due to these features, we interpret the data obtained with some caution.

At the final stage, all computed parameters are combined into a feature vector comprising rhythm-, morphology-, and repolarization-related ECG metrics. The resulting vector is used to identify electrocardiographic patterns associated with glycemic disturbances and is intended to support non-invasive screening rather than clinical diagnosis.

The main stages of digital ECG signal processing and the corresponding parameter calculation methods are summarized in Table 3.

The extracted features form a feature vector f, which is used for subsequent analysis. Key indicators associated with deviations in glycemic status include QTc prolongation, increased QT dispersion, reduced T-wave amplitude, and ST-segment deviation.

To support the physiological relevance of these parameters, common reference ranges and representative deviations reported in the literature for dysglycemia and impaired glucose metabolism are summarized in Table 4.

Thus, the automated algorithm enables real-time quantitative assessment based on ECG data and allows the identification of changes potentially associated with alterations in glycemic status without the use of invasive sensors. All algorithmic modules are implemented within a unified software core of the device (see Section 2.3) and support both local and cloud-based recording of results.

### 2.5. Algorithm Calibration Stage

The calibration stage of the algorithm was carried out prior to the analysis of the main sample and was aimed solely at selecting and fixing global settings for preprocessing, signal quality control, and QT dilution rules. For this purpose, records of additional volunteers (technical calibration group) who were not included in the set of participants presented in Section 3 (including tables and representative examples) were used. After the calibration was completed, all parameters were fixed and then applied to the study data without any changes, which reduces the risk of circularity and optimistic bias.

### 2.6. Expert Verification of the Marking of the End of the T-Wave ($T_{end}$)

To assess the consistency of the automatic determination of the end of the T-wave ($T_{end}$) with expert assessment, a pilot manual check by a cardiologist was performed. For each group (norm, prediabetes, DM2), 16 artifact-free ECG strokes in lead II were selected. The cardiologist manually marked the position of $T_{end}$ for each stroke, then the difference $∆T_{end}=T_{end,cardiologist}-T_{end,auto}$, was calculated, where $T_{end}$ > 0 means a later diagnosis of $T_{end}$ by a cardiologist, ${∆T}_{end}$ < 0 − an earlier one. For each class, we calculated the average displacement of the Mean ($Mean{∆T}_{end}$), mean absolute error ($Mean\left|{∆T}_{end}\right|$),) and standard deviation ${∆T}_{end}$ (SD). The sampling rate was Fs = 500 Hz (1 sample = 2 ms). The obtained indicators are considered as an illustrative assessment of consistency on selected strokes.

## 3. Results

### 3.1. Experimental Sample and Study Conditions

A total of 12 volunteers participated in the study. Among them, 10 participants were classified as conditionally healthy, 1 participant had a previously established diagnosis of type 2 diabetes mellitus, and 1 participant exhibited impaired glucose tolerance (prediabetes). A further 14 volunteers were involved in the algorithm calibration stage.

Prior to ECG acquisition, written informed consent was obtained from each participant. All volunteers were informed in advance about the objectives and procedures of the study. The experiment was conducted in accordance with ethical standards and under the supervision of a cardiologist. All the collected data were anonymized, and the study protocol was reviewed and approved by experts.

In addition, each participant completed a questionnaire containing information on age, sex, height, body mass, level of physical activity, presence of harmful habits (smoking and alcohol consumption), dietary characteristics, fasting glucose level, history of diabetes mellitus and cardiovascular diseases, and follow-up by a cardiologist and/or endocrinologist.

For each participant, a 12-lead ECG recording was performed in both standing and sitting positions for a duration of 1 min. In participants with impaired carbohydrate metabolism (prediabetes and type 2 diabetes mellitus), ECG recordings were repeated at different time points, whereas in healthy volunteers, the recording was performed once. ECG recordings obtained in the standing and sitting positions were processed separately. All QT-related indices (QT, QTc, and QTd) were computed independently for each body position, and no averaging across postures was performed to avoid mixing posture-related autonomic effects. Prior to ECG acquisition, fasting capillary blood glucose levels were measured in all participants using a portable glucometer, which enabled the classification of participants according to glycemic status (normoglycemia, prediabetes, and type 2 diabetes mellitus).

A summary of the characteristics of the study cohort is presented in Table 5.

The obtained experimental cohort had a pilot nature and was formed to enable a preliminary assessment of the feasibility of identifying electrocardiographic features associated with disturbances in carbohydrate metabolism. As shown in Table 5, the study groups differ in age and sex distribution: healthy volunteers are predominantly younger, whereas participants with impaired glucose tolerance and type 2 diabetes mellitus belong to an older age group. Nevertheless, age and gender are the main factors that distort the results of the analysis of ECG markers associated with repolarization; therefore, the presented pilot data set does not allow these factors to be taken into account.

Despite the limited number of participants in the prediabetes and type 2 diabetes mellitus groups, repeated ECG recordings obtained from these individuals under different physiological conditions (standing and sitting positions) provided multiple within-subject measurements, allowing an initial assessment of parameter stability under standardized conditions; however, this does not address between-subject variability and does not enable group-level inference. This approach allows the assessment of the stability of the extracted ECG parameters and their sensitivity to changes in glycemic status.

It should be noted that the present study was not designed to yield statistically generalizable conclusions for the overall population. The primary objective was to identify trends and preliminary patterns in ECG parameter changes associated with impaired carbohydrate metabolism, which may serve as a basis for future investigations involving larger and more balanced cohorts.

The following section provides representative examples of the QT interval-related parameters observed in this pilot dataset. The results are presented in a descriptive form and are not intended for statistical generalization at the group level.

### 3.2. Representative Case Examples of QT-Related Parameters

This section describes the parameters related to the QT interval (QT, QTc, QTd) in representative participants with different glycemic statuses. The analysis focused on QT-related indices, including the QT interval, the heart rate–corrected QT interval (QTc), and QT dispersion (QTd), which are widely regarded as sensitive indicators of metabolic and autonomic disturbances associated with impaired glucose metabolism.

In the conditionally healthy participant, both QTc and QT dispersion values were within physiological reference ranges, indicating preserved electrical homogeneity of myocardial repolarization. In the participant with impaired glucose tolerance, the QTc interval remained within the normal range; however, an increase in QT dispersion was observed. This observation may be consistent with increased heterogeneity of ventricular repolarization in this representative case; however, confounding cannot be excluded in this pilot dataset. In contrast, the participant with type 2 diabetes mellitus demonstrated pronounced QTc prolongation accompanied by a substantial increase in QT dispersion, reflecting more advanced alterations in myocardial repolarization.

The quantitative values of QT, QTc, and QT dispersion parameters for the studied participants are summarized in Table 6.

QTc was calculated using the Bazett correction formula. QT dispersion (QTd) was defined as the difference between the maximum and minimum QT values measured across standard ECG leads.

In the normoglycemic participant, the corrected QT interval (QTc) and QTd were within the reference ranges, indicating preserved electrical homogeneity of the myocardium.

In the participant with impaired glucose tolerance, QTc values remained within normal limits; however, an increase in QT dispersion was observed. This observation may be consistent with increased heterogeneity of ventricular repolarization in this representative case, but we cannot exclude the influence of confounding factors in this pilot dataset.

In the group with type 2 diabetes mellitus, a marked increase in both QTc and QTd was observed, reflecting substantial impairments in ventricular repolarization. The values obtained in our pilot dataset should be considered descriptive and case-based, rather than evidence of “progressive” or stage-dependent changes. In representative cases, repolarization-related parameters (QT/QTc/QTd) may have differed between glycemic status categories; however, these observations require confirmation in a larger, age- and band-matched sample.

### 3.3. Visual Results and Examples of System Operation

**Figure 7:**
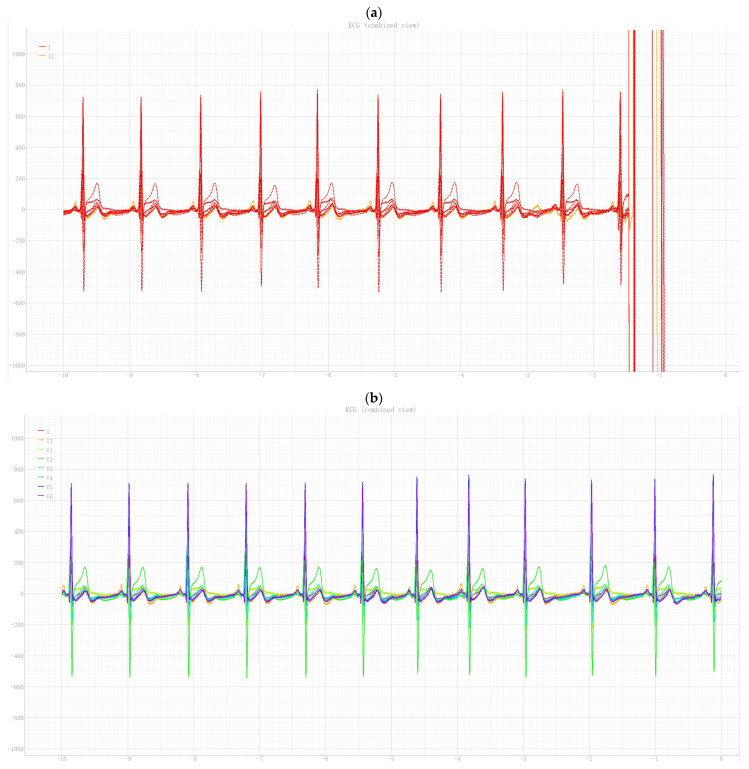
Software interface of the proposed ECG system illustrating signal quality control during data acquisition: (**a**) automatic pause of ECG recording triggered by electrode disconnection (lead-off detection), resulting in temporary suspension of data acquisition and visual notification of signal loss; (**b**) restoration of normal ECG signal visualization and resumption of data acquisition after electrode contact is re-established.

Figure 7 presents the software interface during electrode disconnection and after contact restoration. When an electrode connection is lost, the system automatically pauses ECG recording and visually notifies the user of a signal quality issue. Once contact is restored, data acquisition resumes without loss of previously recorded data. This mechanism prevents distorted signal segments from being included in the analysis and ensures high reliability of the computed parameters.

Upon normal session termination, a controlled recording shutdown mechanism is activated. This mechanism disables all active timers, resets recording and pause status flags, restores the user interface elements to their initial state, and initiates the procedure for saving the accumulated data. This ensures the integrity of the recorded signal and associated metadata regardless of whether automatic pauses occurred during acquisition.

**Figure 8:**
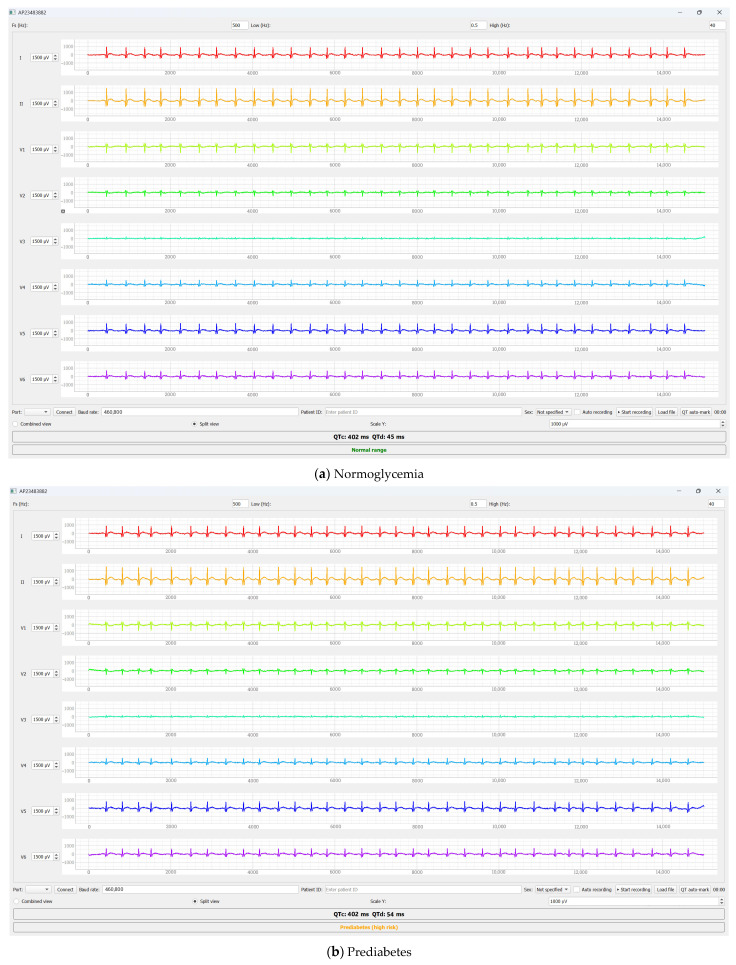

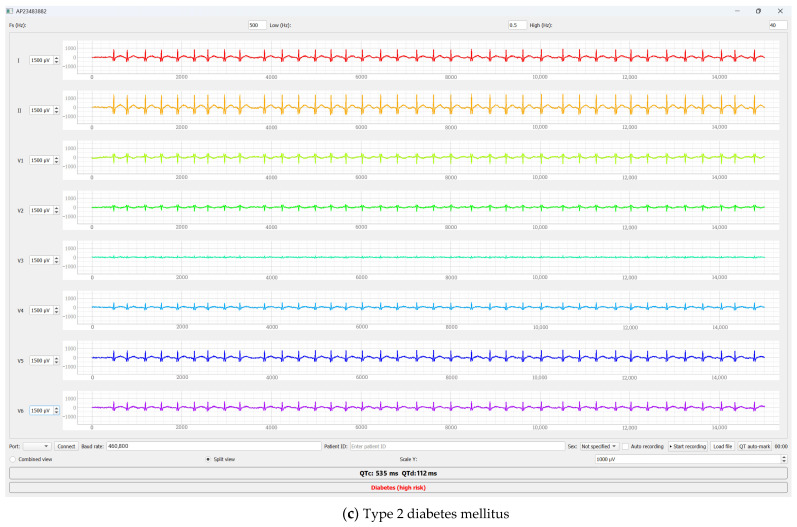
Representative ECG segments illustrating multichannel ECG acquisition and signal quality in participants with different glycemic statuses: (**a**) normoglycemia, (**b**) prediabetes (impaired glucose tolerance), and (**c**) type 2 diabetes mellitus. The signals are shown with identical horizontal and vertical scales (*x*-axis: samples; Fs = 500 Hz (1 sample = 2 ms); *y*-axis: mV).

Figure 8 shows representative multi-channel ECG segments (displayed using the same horizontal and vertical scales in all panels, *x*-axis: counts, Fs = 500 Hz) recorded from participants with different glycemic statuses and illustrates the overall signal quality and morphology with the proposed acquisition configuration.

**Figure 9:**
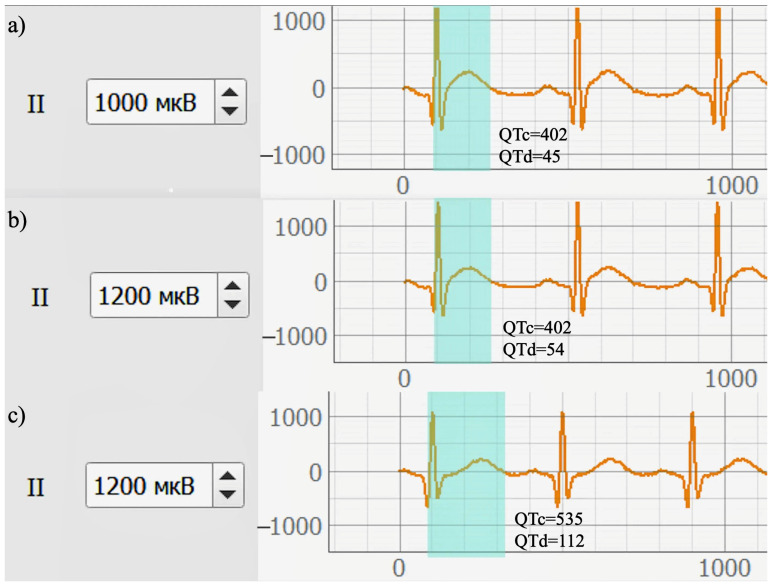
Electrocardiographic segments from lead II with highlighted QT intervals in participants with normoglycemia, prediabetes, and type 2 diabetes mellitus. Representative ECG segments from lead II are shown for: (**a**) a normoglycemic participant; (**b**) a participant with impaired glucose tolerance (prediabetes); and (**c**) a participant with type 2 diabetes mellitus. The shaded area indicates the QT interval measured from the onset of the Q wave to the end of the T wave. The corrected QT interval (QTc) and QT dispersion (QTd) values are numerically indicated for each example. All ECG signals are displayed using the same time and amplitude scales.

A visual illustration of differences in ventricular repolarization is provided in Figure 9, which presents representative ECG segments from lead II for participants with different glycemic statuses. In the participant with impaired glucose tolerance, the QT interval appears prolonged compared with the normoglycemic participant, while the corrected QT interval (QTc) remains within normal limits and is accompanied by increased QT dispersion.

In contrast, the participant with type 2 diabetes mellitus exhibits a pronounced QTc prolongation along with noticeable alterations in the morphology of the repolarization phase. These visual observations are consistent with the quantitative results presented in Table 6 is consistent with the descriptive differences associated with the QT interval observed in these representative samples; however, causal attribution to glycemic status is not possible in this pilot dataset.

As shown in Figure 9, the normoglycemic participant exhibits QTc and QTd values within the reference ranges. In the participant with prediabetes, QTc remains within normal limits; however, an increase in QT dispersion is observed, which may indicate enhanced heterogeneity of ventricular repolarization processes. In the case of type 2 diabetes mellitus, a pronounced increase in both QTc and QTd is evident, reflecting substantial impairments in myocardial electrical stability.

QTc and QTd values were calculated as described in Section 2 using standard QT correction and dispersion definitions.

### 3.4. Consistency of Automatic $T_{end}$ Markup with Expert Evaluation

A pilot expert review showed that, on average, the automated algorithm and the cardiologist determined the end of the T-wave almost identically in all three groups ($Mean{∆T}_{end}$ of about −1 to −2 ms). However, the magnitude of error (MAE) for individual beats was higher in participants with prediabetes and diabetes compared to the normal group, possibly due to the more variable T-wave morphology and less clear definition of its end in individual recording fragments. Detailed agreement indicators are presented in Table 7.

## 4. Discussion

In the present study, the feasibility of identifying electrocardiographic changes associated with impaired glucose metabolism was evaluated based on the analysis of multichannel electrocardiographic recordings. Particular attention was paid to ventricular repolarization parameters, as these indices have been shown in the literature [33,34] to be sensitive to metabolic and autonomic alterations observed in prediabetes and type 2 diabetes mellitus.

It is important to emphasize a limitation of interpretation: due to the marked age and gender imbalance between the normal group and isolated cases of prediabetes and T2DM, the observed differences in QT/QTc/QTd cannot be attributed solely to glycemic status. Therefore, the results should be viewed as descriptive, hypothesis-generating observations, rather than as evidence of stage-dependent metabolic changes.

Within this pilot dataset, repolarization-related parameters (QT, QTc, and QTd) may have differed between glycemic status categories in some representative cases. These observations are descriptive and hypothesis-generating in nature and require confirmation in larger age- and band-matched cohorts with appropriate statistical modeling and control for confounding factors. In the normoglycemic participant, the corrected QT interval (QTc) and QT dispersion (QTd) remained within physiological reference ranges, indicating preserved electrical homogeneity of the myocardium. In the participant with impaired glucose tolerance, QTc values remained within normal limits; however, an increase in QT dispersion was observed. This is a descriptive, hypothesis-generating observation of increased repolarization heterogeneity in this representative case, without implying any temporal sequence relative to QTc prolongation. In contrast, the participant with type 2 diabetes mellitus exhibited a pronounced increase in both QTc and QTd, reflecting more profound impairments in myocardial electrical stability. Potential confounding factors cannot be excluded in this pilot dataset.

Potential pathophysiological mechanisms of repolarization changes, such as the contribution of autonomic dysfunction, ion channel remodeling, oxidative stress, and microcirculatory disorders, are widely discussed in the literature, but this pilot dataset does not allow us to confirm the mechanisms or draw conclusions about the stage progression. In our work, these provisions are provided solely as a context from the literature and as a justification for the need for further clinical validation.

A visual illustration of the identified alterations is provided in Figure 9, which presents representative ECG segments from lead II for participants with different glycemic statuses. In contrast to Figure 8, which primarily demonstrates signal quality and features of multichannel ECG acquisition, Figure 9 specifically emphasizes differences in the QT interval and the corresponding QTc and QTd values. The visual observations shown in this figure are in good agreement with the quantitative results reported in Table 6.

An important advantage of the proposed approach is the implementation of automated signal quality control mechanisms. The use of electrode detachment detection and recording process management allows corrupted ECG segments to be excluded from further analysis. This is particularly critical for the assessment of QT and QTc intervals, as the presence of artifacts can lead to substantial errors in waveform boundary identification. The robustness of the implemented signal processing algorithm enhances the reliability of the extracted parameters and supports the suitability of the system for further development and scaling.

Several limitations of this study should be acknowledged. First, the study has a pilot design and includes a limited number of participants with prediabetes and type 2 diabetes mellitus, which precludes statistically generalizable conclusions. Second, differences in age and sex among participants may influence repolarization parameters and should be more rigorously controlled in future studies. Third, although key repolarization indices, including QTc and QT dispersion, were analyzed, other potentially informative markers—such as the Tp–e interval, Tp–e/QT ratio, and heart rate variability indices—were not evaluated and warrant further investigation.

Despite these limitations, the results obtained should be interpreted as pilot, hypothesis-generating observations demonstrating the technical feasibility of recording a 12-channel ECG and calculating QT parameters in a wearable format. Any assumptions about the “sequence” or stage-dependent dynamics of changes require separate clinical validation in extended cohorts with laboratory confirmation of glycemic status and control of confounding factors. The observed configuration of indicators in individual examples (for example, changes in QTd with QTc retained in one case and increases in QTc and QTd in the other) should not be interpreted as a proven step sequence. This is just a hypothesis that needs to be tested on extended, age- and gender-comparable cohorts with laboratory verification of glycemic status and a correct statistical model. Further validation will require comparison with expert markup and benchmarking using existing methods.

The QT/QTc indices and the QT variance (QTd) are not specific for carbohydrate metabolism disorders and may change with coronary heart disease, structural pathology of the myocardium, electrolyte imbalance, under the influence of drug therapy (for example, beta-blockers, antiarrhythmic drugs), as well as with changes in autonomic tone. Since these factors were not systematically evaluated or controlled within the framework of the pilot set, the observed differences should be interpreted solely as preliminary, descriptive, and hypothetical. In subsequent studies, control groups with cardiovascular diseases and standardized clinical characteristics of participants are needed.

The inclusion of wireless data transmission is functionally motivated rather than merely technical. The wearable configuration allows ECG acquisition in different postures without physical tethering to a stationary workstation, reducing mechanical constraints and potential cable-induced artifacts. In future screening-oriented applications, wireless transmission would support centralized storage and remote expert review, particularly in resource-limited settings.

## 5. Conclusions

In this work, we examined whether multilead ECG analysis could reveal electrophysiological features potentially related to impaired glucose metabolism. The upgraded wearable 12-lead system provided stable signal acquisition and allowed consistent extraction of ventricular repolarization parameters, including QT, QTc, and QT dispersion, under standardized recording conditions. In the pilot dataset, individual cases differed in QT-related indices depending on glycemic status. QTc and QTd were within reference limits in the normoglycemic participant. In the case of impaired glucose tolerance, QTc did not show marked prolongation, whereas QT dispersion was increased. In the participant with type 2 diabetes mellitus, both QTc and QTd were elevated. These findings cannot be generalized and should not be interpreted as stage-dependent patterns; the cohort was small and demographically unbalanced, and the influence of age, sex, autonomic tone, and comorbidities cannot be excluded.

QT correction was performed using Bazett’s formula. Diagnostic performance metrics were not calculated at this stage, as reliable estimation requires larger cohorts with biochemical confirmation of glycemic status. It should also be noted that QT-derived markers are not specific to metabolic disorders and may be influenced by cardiovascular disease, medication, or electrolyte imbalance. The study demonstrates that synchronized wearable 12-lead acquisition combined with systematic multilead analysis is technically feasible and reproducible. The present results should therefore be regarded as preliminary, forming a basis for larger clinically characterized investigations aimed at clarifying the potential role of ECG-derived markers in non-invasive metabolic risk assessment.

## Figures and Tables

**Table 1:** Mathematical formulation of the twelve standard electrocardiographic leads.

№	Leads	Formula	Description
Wilson Central Terminal (WCT)			
$WCT=\frac{RA+LA+LL}{3}$			Wilson’s central terminal (WCT) is the average electrical potential of the right arm (RA), left arm (LA), and left leg (LL) electrodes
Standard Limb Leads			
1	Lead I	I = LA − RA	Standard limb leads represent the potential differences between limb electrodes
2	Lead II	II = LL − RA	
3	Lead III	III = LL − LA	
Augmented Limb Leads			
4	aVR (augmented right arm lead)	aVR = RA − $\frac{LA+LL}{2}$	Augmented limb leads (aVR, aVL, and aVF) are unipolar leads whose reference potential is the Wilson central terminal
5	aVL (augmented left arm lead)	aVL = LA − $\frac{RA+LL}{2}$	
6	aVF (augmented left foot lead)	aVF = LL − $\frac{RA+LA}{2}$	
Precordial (Chest) Leads			
7	V1 precordial lead	$V_{1}=V_{1electrode}$ − WCT	Precordial leads (V1–V6) are unipolar leads referenced to the Wilson central terminal
8	V2 precordial lead	$V_{2}=V_{2electrode}$ − WCT	
9	V3 precordial lead	$V_{3}=V_{3electrode}$ − WCT	
10	V4 precordial lead	$V_{4}=V_{4electrode}$ − WCT	
11	V5 precordial lead	$V_{5}=V_{5electrode}$ − WCT	
12	V6 precordial lead	$V_{6}=V_{6electrode}$ − WCT	

**Table 2:** General structure of the data packet transmitted by the device.

Field	Size (Bytes)	Description
Header	3	Fixed synchronization word (0 × AA 0 × FF 0 × F1) indicating the start of a data packet.
Length	1	Number of payload bytes (fixed at 0 × 22 = 34 in the current implementation).
Payload (ECG data)	32	Eight ECG channels (I, II, and V1–V6) encoded as 32-bit signed integers (int32 and little-endian).
Lead-off flags	2	Bitwise indicators of electrode disconnection, where each bit corresponds to loss of contact on a specific channel.
Checksums	2	Checksum 1 represents the sum of all packet bytes modulo 256; Checksum 2 is a cumulative checksum used for error detection.

**Table 3:** Main stages of digital ECG signal processing and parameter extraction.

	Stage	Description	Formula/Method
1	Signal decoding	Conversion of raw digital values into microvolts based on ADS1298 parameters	$U=raw\times 0.286\left[\mu V\right]$
2	DC offset removal	Removal of the DC component of the signal	High-pass filter: $f_{s}\approx 0.5\;Hz$
3	Noise suppression	Suppression of power-line and muscle interference	Notch filter (50 Hz); LPF: $f_{s}\approx 125\;Hz$
4	R-peak detection	Peak detection with a minimum inter-peak interval	Peak detection algorithm: ∆t>0.4 s
5	RR and HR calculation	Inter-peak intervals and heart rate	$HR=\frac{60}{{RR}_{avg}},SDNN=\sqrt{\frac{1}{N-1}\sum {\left({RR}_{i}-\bar{RR}\right)}^{2}}$
6	Waveform morphology	Measurement of P-wave, QRS complex, and T-wave durations and amplitudes, and ST-segment deviation	$T_{amp}=T_{peak}-baseline;$ ${ST}_{shift}={ST}_{avg}-{PQ}_{baseline}$
7	QT and QTc intervals	Calculation of the QT interval and its heart rate correction	$QTc=\frac{QT}{\sqrt{RR}};$ ${QT}_{d}={QT}_{max}-{QT}_{min}$
8	Feature vector	Formation of a set of assessment metrics for evaluating the patient’s condition	$f=\left\{QTc,{QT}_{d},T_{amp},{ST}_{shift},HR,\ldots \right\}$

**Table 4:** Reference ranges and representative deviations of ECG parameters reported in dysglycemia and diabetes-related metabolic disturbances.

Parameter	Normal Range	Alterations Reported in Dysglycemia/Diabetes-Related Metabolic Disturbances	Physiological Relevance
RR interval	600–1000 ms (60–100 bpm)	Shortening or prolongation (tachycardia/bradycardia)	Association with heart rate and autonomic regulation
SDNN	>50 ms	Decrease	Marker of heart rate variability
QT interval	350–440 ms	Prolongation	Sensitivity to metabolic alterations, arrhythmia risk
QTc (Bazett)	<450 ms (men), <470 ms (women)	Prolongation	Indicator associated with arrhythmic risk and altered glucose metabolism
QT dispersion (QTd)	<50 ms	Increase	Heterogeneity of ventricular repolarization
T-wave amplitude	0.1–0.5 mV	Reduction	Indicator of ionic imbalance
ST-segment deviation	≤±0.1 mV	Possible depression	Ischemic and electrolyte-related alterations

**Table 5:** Characteristics of the study participants.

Participant Group	Number (n)	Sex (M/F)	Age, Years (M ± SD)	Fasting Glucose (mmol/L)	Glycemic Status	Body Position During ECG Recording
Healthy	10	7/3	24 ± 3.2	4.6 ± 0.4	No diabetes	Single recording, standing and sitting
Prediabetes	1	1/0	56	6.0	Impaired glucose tolerance	Repeated recordings, standing/sitting
Type 2 diabetes mellitus	1	0/1	53	7.8	Type 2 diabetes mellitus	Repeated recordings, standing/sitting

**Table 6:** Representative QT, QTc, and QT dispersion values in the pilot dataset.

Group	QT Interval (ms)	QTc (ms)	QT Dispersion, QTd (ms)	Comment
Healthy	~380	402	45	Normal ventricular repolarization
Prediabetes	~390	402	54	Increased QT dispersion with preserved QTc
Type 2 diabetes mellitus	~480	535	112	Marked QTc prolongation and increased repolarization heterogeneity

**Table 7:** Beat-level agreement for T-wave end ($T_{end}$) placement: automated vs. cardiologist (Lead II, Fs = 500 Hz).

Group	n (beats)	$\mathrm{Mean}\;{∆T}_{end}$ (ms)	MAE (ms)	SD (ms)
Normal	16	−0.8	10.5	13.1
Prediabetes	16	−1.2	20.8	18.2
Diabetes	16	−1.8	24.0	20.3

## Data Availability

The data supporting the findings of this study include anonymized multi-lead electrocardiographic (ECG) recordings, derived quantitative ECG parameters, and aggregated questionnaire data describing basic demographic and clinical characteristics of the participants (e.g., age, sex, anthropometric parameters, and glycemic status), as reported in Section 3.1. All questionnaire data were collected without direct personal identifiers and were used only in a summarized form. Due to the small sample size and ethical considerations related to participant privacy, the datasets are not publicly available. However, the data can be made available from the corresponding author upon reasonable request.

## Supplementary Materials

The following supporting information can be downloaded at: https://www.mdpi.com/article/10.3390/s26051598/s1, Figure S1: Schematic electrical circuit of the device.

## Abbreviations

The following abbreviations are used in this manuscript:

ACCF	American College of Cardiology Foundation
AHA	American Heart Association
ECG	Electrocardiogram
QTc	Corrected QT interval
QTd	QT dispersion
SDNN	Standard Deviation of Normal-to-Normal (NN) intervals
T2DM	Type 2 Diabetes Mellitus
WCT	Wilson Central Terminal
