# Supplementary figures and images for: Development and Pilot Evaluation of a Wearable 12-Lead ECG System for Multilead Feature Analysis in Individuals with Different Glycemic Status
Source: Sensors (Basel). 2026 Mar 4;26(5):1598. doi: 10.3390/s26051598 (PMC12987027; doi:10.3390/s26051598)

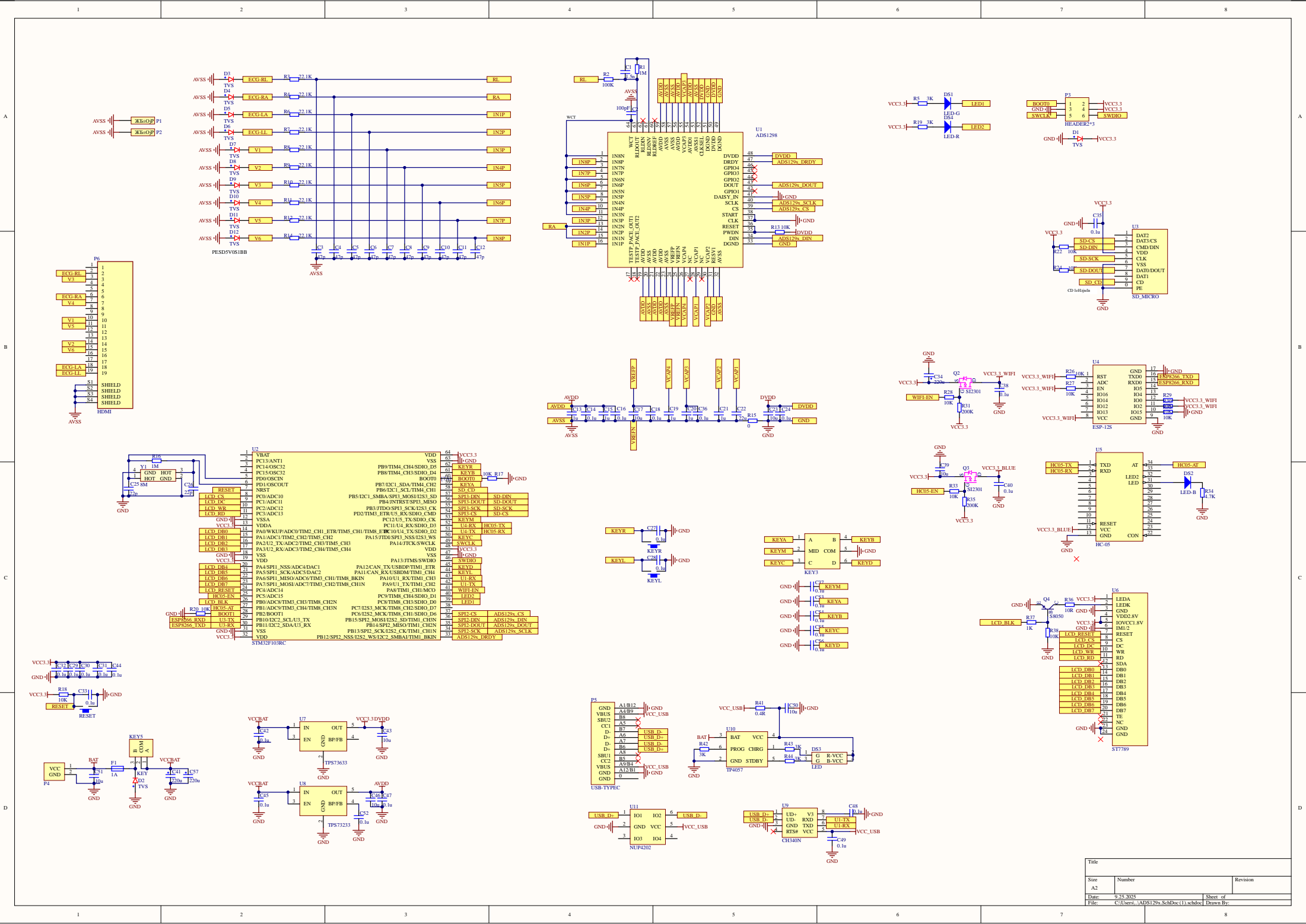

|       |                                        |           |          |
|-------|----------------------------------------|-----------|----------|
| Title |                                        |           |          |
| Size  | Number                                 |           | Revision |
| A2    |                                        |           |          |
| Date: | 9.25.2025                              | Sheet of  |          |
| File: | C:\Users\... \ADS129x.SchDoc(1).schdoc | Drawn By: |          |

Supplement: Supplementary file 1 [file sensors-26-01598-s001.zip › sensors-4157356-supplementary.pdf]
